# Impact of Concomitant Thiopurine on the Efficacy and Safety of Filgotinib in Patients with Ulcerative Colitis: Post Hoc Analysis of the Phase 2b/3 SELECTION Study

**DOI:** 10.1093/ecco-jcc/jjad201

**Published:** 2023-11-29

**Authors:** Kenji Watanabe, Laurent Peyrin-Biroulet, Silvio Danese, Yasushi Fujitani, Margaux Faes, Alessandra Oortwijn, James O Lindsay, Gerhard Rogler, Toshifumi Hibi

**Affiliations:** Department of Internal Medicine for Inflammatory Bowel Disease, University of Toyama, Toyama, Japan; Department of Gastroenterology, CHRU-Nancy, University of Lorraine, Nancy, France; Inserm, NGERE, University of Lorraine, Nancy, France; Gastroenterology and Endoscopy, IRCCS Hospital San Raffaele, Milan, Italy; Vita-Salute San Raffaele University, Milan, Italy; Gilead Sciences K.K., Tokyo, Japan; Galapagos GmbH, Basel, Switzerland; Galapagos NV, Oegstgeest, Netherlands; Centre for Immunobiology, Blizard Institute, Barts and the London School of Medicine, Queen Mary University of London, London, UK; Department of Gastroenterology and Hepatology, University Hospital of Zurich, University of Zurich, Zurich, Switzerland; Center for Advanced IBD Research and Treatment, Kitasato Institute Hospital, Kitasato University, Tokyo, Japan

**Keywords:** Ulcerative colitis, thiopurine, filgotinib

## Abstract

**Background and Aims:**

SELECTION is the first study to assess the impact of concomitant thiopurine and other immunomodulator [IM] use on the efficacy and safety of a Janus kinase inhibitor, filgotinib, in patients with ulcerative colitis.

**Methods:**

Data from the phase 2b/3 SELECTION study were used for this post hoc analysis. Patients were randomised [2:2:1] to two induction studies [biologic-naive, biologic-experienced] to filgotinib 200 mg, 100 mg, or placebo. At Week 10, patients receiving filgotinib were re-randomised [2:1] to continue filgotinib or to switch to placebo until Week 58 [maintenance]. Outcomes were compared between subgroups with and without concomitant IM use.

**Results:**

At Week 10, similar proportions of patients in the +IM and −IM groups treated with filgotinib 200 mg achieved Mayo Clinic Score [MCS] response [biologic-naive: 65.8% vs 66.9%; biologic-experienced: 61.3% vs 50.5%] and clinical remission [biologic-naive: 26.0% vs 26.2%; biologic-experienced: 11.3% vs 11.5%]. At Week 58, similar proportion of patients in the +IM and −IM groups treated with filgotinib 200 mg achieved MCS response [biologic-naive: 74.2% vs 75.0%; biologic-experienced: 45.5% vs 61.4%] and clinical remission [biologic-naive: 51.6% vs 47.4%; biologic-experienced: 22.7% vs 24.3%]. The probability of protocol-specified disease worsening during the maintenance study in patients treated with filgotinib 200 mg did not differ between +IM and −IM groups [*p* = 0.6700]. No differences were observed in the incidences of adverse events between +IM and −IM groups in the induction/maintenance studies.

**Conclusions:**

The efficacy and safety profiles of filgotinib treatment in SELECTION did not differ with or without concomitant IM use.

**ClinicalTrials.gov identifier:**

NCT02914522.

## 1. Introduction

Ulcerative colitis [UC] is a chronic inflammatory disease of the colonic mucosa that typically begins in the rectum.^1^ Therapies approved for the treatment of moderately to severely active UC include systemic corticosteroids, thiopurines, and other immunomodulators [IMs], as well as targeted biologics, such as tumour necrosis factor [TNF] antagonists [infliximab, adalimumab, and golimumab], the anti-α4β7 integrin antibody vedolizumab, and the anti-interleukin [IL]-12/IL-23 p40 antibody ustekinumab.^2^ Importantly, corticosteroid-associated adverse events [AEs] continue to be problematic and many patients with UC respond sub-optimally to targeted biologics.^3,4^ Therefore, additional treatment options are needed.

Janus kinase [JAK] inhibitors are small-molecule drugs that interfere with the JAK/signal transducers and activator of transcription [STAT] signalling pathway. JAK inhibitors modify cytokine signalling and reduce downstream inflammation associated with UC via inhibition of one or more intracellular kinases involved in the JAK/STAT pathway [JAK1, JAK2, JAK3, and tyrosine kinase 2].^5^ Filgotinib is a once-daily, oral JAK1 preferential inhibitor approved in the European Union, Japan, and the UK for the treatment of moderately to severely active UC in patients who have had an inadequate response, loss of response, or are intolerant to either conventional or biologic therapy.^6,7^ These indications are based on results from the global phase 2b/3, double-blind, randomised, placebo-controlled SELECTION study.^8^ The SELECTION study reported statistically significantly higher rates of clinical remission with filgotinib 200 mg than with placebo, during induction and maintenance phases in both biologic-experienced and biologic-naive patients.^8^ Filgotinib 200 mg was well tolerated in SELECTION, with similar incidences of serious AEs [SAEs] occurring in the filgotinib and placebo treatment groups.^8^

The impact of concomitant IMs, such as azathioprine [AZA], 6-mercaptopurine [MP], and methotrexate, on the efficacy and safety of targeted biologic treatment, are of particular interest in the treatment of UC and Crohn’s disease.^9–14^ In the randomised, double-blind, phase 3 UC SUCCESS trial, the combination of infliximab and AZA significantly improved rates of corticosteroid-free remission compared with either drug alone in TNF antagonist-naive patients with moderate to severe UC.^9^ In addition, a recent exploratory analysis suggested that concomitant IM use may be beneficial for maintaining clinical remission in Japanese patients with moderately to severely active UC treated with vedolizumab.^15^ The study indicated that, at Week 60, mucosal healing was achieved in a higher proportion of patients treated with vedolizumab and a concomitant IM [77.3%] than in those without a concomitant IM [47.4%].^15^ Importantly, the concomitant use of any IM with a JAK inhibitor is yet to be evaluated in patients with inflammatory bowel disease, and filgotinib is the only JAK inhibitor approved for use with concomitant IMs in UC.^6,16,17^

Here, we conducted a post hoc analysis of the SELECTION study to assess the impact of protocol-accepted concomitant IM use on the efficacy and safety of filgotinib treatment in patients with moderately to severely active UC.

## 2. Materials and Methods

### 2.1. SELECTION study design and participants

Data for this post hoc analysis were obtained from the phase 2b/3 randomised, double-blind, placebo-controlled SELECTION study [ClinicalTrials.gov identifier: NCT02914522], for which details have been reported previously [Supplementary Figure 1].^8^ The SELECTION protocol was reviewed and approved by a study-site specific independent ethics committee or institutional review board. The study was carried out in accordance with the Declaration of Helsinki and the International Conference on Harmonisation for Good Clinical Practice Guidelines. All study participants provided informed consent.

Patients aged 18–75 years with moderately to severely active UC (Mayo endoscopy sub-score ≥ 2, rectal bleeding sub-score ≥ 1, stool frequency sub-score ≥ 1, physician’s global assessment sub-score ≥ 2, and Mayo Clinic Score [MCS] 6–12), with a documented diagnosis of UC for at least 6 months before enrolment, were randomised [2:2:1] to one of two induction studies [A and B] to receive filgotinib 200 mg, filgotinib 100 mg, or placebo for 11 weeks. Induction study A included biologic-naive patients, defined as those without previous use of a TNF antagonist [eg, infliximab, adalimumab, golimumab, certolizumab, or a biosimilar agent] or vedolizumab. Induction study B included biologic-experienced patients, defined as those with previous inadequate clinical response, loss of response, or intolerance to a TNF antagonist or vedolizumab, and without any use of these agents for at least 8 weeks before screening.

At Week 10, patients receiving filgotinib who achieved clinical remission [Mayo endoscopic sub-score 0 or 1, rectal bleeding sub-score 0, and ≥ 1-point decrease from baseline in stool frequency sub-score to achieve a sub-score of 0 or 1] or MCS response (≥ 3-point [and at least 30%] reduction from baseline in MCS accompanied by a ≥ 1-point decrease from baseline in rectal bleeding sub-score, or a Week 10 rectal bleeding sub-score of 0 or 1) were re-randomised [2:1] to continue assigned filgotinib or to switch to placebo for 47 weeks [maintenance study] [Supplementary Figure 1].

Participants eligible for inclusion in SELECTION included those already receiving an IM [AZA, MP, or methotrexate]. Continued, concomitant use of an IM with filgotinib during SELECTION was allowed if the dose was stable at least 4 weeks before and 10 weeks after randomisation. No IM dosing guidance was given for the maintenance study. No other restrictions regarding the use of IMs were applied during the SELECTION study. Corticosteroid tapering was initiated at Week 14 of the SELECTION study, as reported previously.^8^

### 2.2. Post hoc analyses

Efficacy outcomes included in the current analysis were clinical remission, MCS response, endoscopic remission [centrally read Mayo endoscopic sub-score of 0], and Geboes histological remission based on the Geboes Score^18^ (centrally diagnosed no or mild increase in chronic inflammatory infiltrate in lamina propria, no neutrophils in lamina propria or epithelium, and erosion, ulceration, or granulation tissues [Grade 0 of ≤ 0.3, Grade 1 of ≤ 1.1, Grade 2a of ≤ 2A.3, Grade 2b of 2B.0, Grade 3 of 3.0, Grade 4 of 4.0, and Grade 5 of 5.0]).

The proportions of patients achieving efficacy outcomes at Week 10 [induction studies A and B] and Week 58 [maintenance study] in the filgotinib 200 mg, filgotinib 100 mg, and placebo treatment groups were calculated, along with corresponding 95% confidence intervals [CIs], for patients with vs without concomitant IM use [+IM vs −IM]. Non-stratified risk differences [RDs] with 95% CIs were used to compare efficacy outcomes for filgotinib vs placebo within the +IM and −IM groups. Probabilities of protocol-specified disease worsening during the 47-week maintenance study were estimated in the filgotinib 200 mg, filgotinib 100 mg, and placebo treatment groups by concomitant IM use using the Kaplan–Meier method. Time to protocol-specified disease worsening was defined as an increase in partial MCS of 3 points or more to at least 5 points from Week 10 over two consecutive visits, or an increase to 9 points over two consecutive visits if the value at Week 10 was over 6. Differences in the time to protocol-specific disease worsening curves were assessed for the +IM and −IM groups using the log-rank test. Cox proportional hazard models were fitted with treatment arms as the independent variable to estimate hazard ratios [HRs] for +IM and −IM subgroups. Proportional hazard assumptions were met by checking Schoenfeld residuals.

Incidences of treatment-emergent AEs were summarised at Week 10 [induction studies A and B combined] and Week 58 [maintenance study] by treatment group and concomitant IM use. AEs of interest included all infections and infestations, serious infections, herpes zoster infections, and pulmonary embolism. AEs were graded using the modified Common Terminology Criteria for Adverse Events [CTCAE], version 4.03. If a CTCAE criterion did not exist, the maximum intensity of the AE was described as Grade 1 [mild], Grade 2 [moderate], Grade 3 [severe], Grade 4 [life-threatening], or Grade 5 [fatal]. Safety analyses and baseline patient characteristics were analysed using descriptive statistics.

All post hoc statistical analyses were carried out using R [version 4.2.2].^19^

## 3. Results

### 3.1. Baseline characteristics and concomitant IM use

Baseline patient characteristics by induction study [A and B] and study treatment are summarised by concomitant IM use in Table 1, and by type of concomitant IM use in Supplementary Table 1. In total, 352 [26.1%] of 1348 patients from induction studies A [biologic-naive] and B [biologic-experienced] combined were receiving an IM at baseline. Of these patients, 288 [81.8%] received AZA, 38 [10.8%] received MP, and 26 [7.4%] received methotrexate [Supplementary Table 3].

**Table 1:** Baseline patient characteristics in induction studies A and B by concomitant IM use.

	Induction study A: biologic-naive patients						Induction study B: biologic-experienced patients					
	FIL 200 mg		FIL 100 mg		PBO		FIL 200 mg		FIL 100 mg		PBO	
	+IM^a^ [*n* = 73]	−IM [*n* = 172]	+IM^a^ [*n *= 82]	−IM [*n* = 195]	+IM^a^ [*n* = 41]	−IM *[n* = 96]	+IM^a^ [*n* = 62]	−IM [*n *= 200]	+IM^a^ [*n* = 62]	−IM [*n *= 223]	+IM^a^ [*n* = 32]	−IM [*n* = 110]
Age, years, mean [SD]	42.2 [12.6]	42.4 [13.3]	42.6 [12.4]	42.2 [13.6]	40.7 [11.5]	41.5 [13.4]	40.8 [15.6]	44.0 [13.7]	40.6 [13.3]	43.6 [14.5]	39.2 [15.0]	45.9 [14.6]
Female sex, *n* [%]	31 [42.5]	91 [52.9]	38 [46.3]	82 [42.1]	14 [34.1]	36 [37.5]	29 [46.8]	85 [42.5]	18 [29.0]	81 [36.3]	10 [31.2]	46 [41.8]
Duration of UC, years, mean [SD]	6.8 [5.4]	7.3 [7.4]	7.1 [8.5]	6.5 [6.9]	5.5 [4.2]	6.8 [8.4]	8.7 [6.7]	10.2 [7.9]	8.1 [6.7]	10.2 [7.2]	9.3 [7.6]	10.4 [8.4]
Total MCS, mean [SD]	8.4 [1.2]	8.7 [1.3]	8.7 [1.4]	8.5 [1.5]	8.7 [1.3]	8.7 [1.4]	9.2 [1.6]	9.2 [1.3]	9.3 [1.5]	9.3 [1.3]	9.1 [1.4]	9.3 [1.4]
Mayo endoscopic score of 3, *n* [%]	45 [61.6]	88 [51.2]	56 [68.3]	103 [52.8]	25 [61.0]	51 [53.1]	53 [85.5]	150 [75.0]	51 [82.3]	171 [76.7]	26 [81.2]	85 [77.3]
C-reactive protein, mg/L, mean [SD]	11.6 [24.6]	7.4 [10.9]	8.8 [22.6]	7.3 [14.7]	4.6 [6.7]	6.3 [7.9]	10.2 [12.0]	12.8 [15.6]	8.9 [15.3]	12.5 [18.6]	7.4 [8.9]	15.9 [26.9]
Faecal calprotectin, μg/g, mean [SD]	1612.8 [2074.4]	2246.0 [2827.7]	2041.5 [3934.1]	1983.2 [3230.7]	2473.7 [3715.2]	2125.8 [2507.1]	2070.8 [3662.6]	3078.7 [4174.2]	2035.0 [3094.5]	2292.8 [3099.8]	1470.5 [1264.5]	2756.1 [3938.1]
Concomitant use of systemic corticosteroids only, *n* [%]	0 [0]	54 [31.4]	0 [0]	67 [34.4]	0 [0]	34 [35.4]	0 [0]	94 [47.0]	0 [0]	103 [46.2]	0 [0]	51 [46.4]
Concomitant use of IMs only, *n* [%]	53 [72.6]	0 [0]	63 [76.8]	0 [0]	33 [80.5]	0 [0]	34 [54.8]	0 [0]	34 [54.8]	0 [0]	21 [65.6]	0 [0]
Concomitant use of systemic corticosteroids and IMs, *n* [%]	20 [27.4]	0 [0]	19 [23.2]	0 [0]	8 [19.5]	0 [0]	28 [45.2]	0 [0]	28 [45.2]	0 [0]	11 [34.4]	0 [0]
Prednisone-equivalent dose, mg/day, median [Q1–Q3]	17.5 [10.0–30.0]	20.0 [10.0–25.0]	15.0 [8.8–20.0]	20.0 [10.0–25.8]	20.0 [10.0–22.5]	20.0 [15.0–30.0]	15.0 [10.0–20.0]	15.0 [10.0–20.0]	20.0 [8.8–20.0]	20.0 [10.0–20.0]	15.0 [7.5–20.0]	20.0 [10.0–20.0]
Prior use of at least one TNF antagonist, *n* [%]	0 [0]	0 [0]	1 [1.2]	1 [0.5]	0 [0]	0 [0]	59 [95.2]	183 [91.5]	57 [91.9]	209 [93.7]	30 [93.8]	100 [90.9]
Prior use of vedolizumab, *n* [%]	0 [0]	0 [0]	0 [0]	1 [0.5]	0 [0]	0 [0]	29 [46.8]	135 [67.5]	26 [41.9]	119 [53.4]	17 [53.1]	68 [61.8]
Prior use of at least one TNF antagonist and vedolizumab, *n* [%]	0 [0]	0 [0]	0 [0]	1 [0.5]	0 [0]	0 [0]	26 [41.9]	121 [60.5]	22 [35.5]	106 [47.5]	15 [46.9]	61 [55.5]
Prior failure of at least one TNF antagonist, *n* [%]	0 [0]	0 [0]	0 [0]	1 [0.5]	0 [0]	0 [0]	50 [80.6]	168 [84.0]	55 [88.7]	196 [87.9]	26 [81.2]	94 [85.5]
Prior failure of vedolizumab, *n* [%]	0 [0]	0 [0]	0 [0]	1 [0.5]	0 [0]	0 [0]	27 [43.5]	121 [60.5]	24 [38.7]	108 [48.4]	16 [50.0]	60 [54.5]

FIL, filgotinib; IM, immunomodulator; MCS, Mayo Clinic Score; PBO, placebo; Q, quartile; SD, standard deviation; TNF, tumour necrosis factor; UC, ulcerative colitis.

^a^+IM includes azathioprine, 6-mercaptopurine, or methotrexate.

At baseline, biologic-experienced patients had a longer mean UC duration than biologic-naive patients [8.1–10.4 years and 5.5–7.3 years, respectively], as well as more severe UC based on mean MCS scores [9.1–9.3 and 8.4–8.7], and more frequent use of an IM in combination with steroids [34.4–45.2% and 19.5–27.4%] [Table 1]. Baseline characteristics between the +IM and −IM groups were generally well balanced.

Baseline patient characteristics in the maintenance study by study treatment and concomitant IM use are summarised in Supplementary Table 2. In total, 145 [21.8%] of 664 patients from the maintenance study [safety analysis set] were receiving a concomitant IM. Of these patients, 119 [82.1%] received AZA, 16 [11.0%] received MP, and 10 [6.9%] received methotrexate [Supplementary Table 4]. Maintenance study baseline characteristics between the +IM and −IM groups were generally well balanced.

### 3.2. Efficacy outcomes with filgotinib induction with or without IM

**Figure 1:**
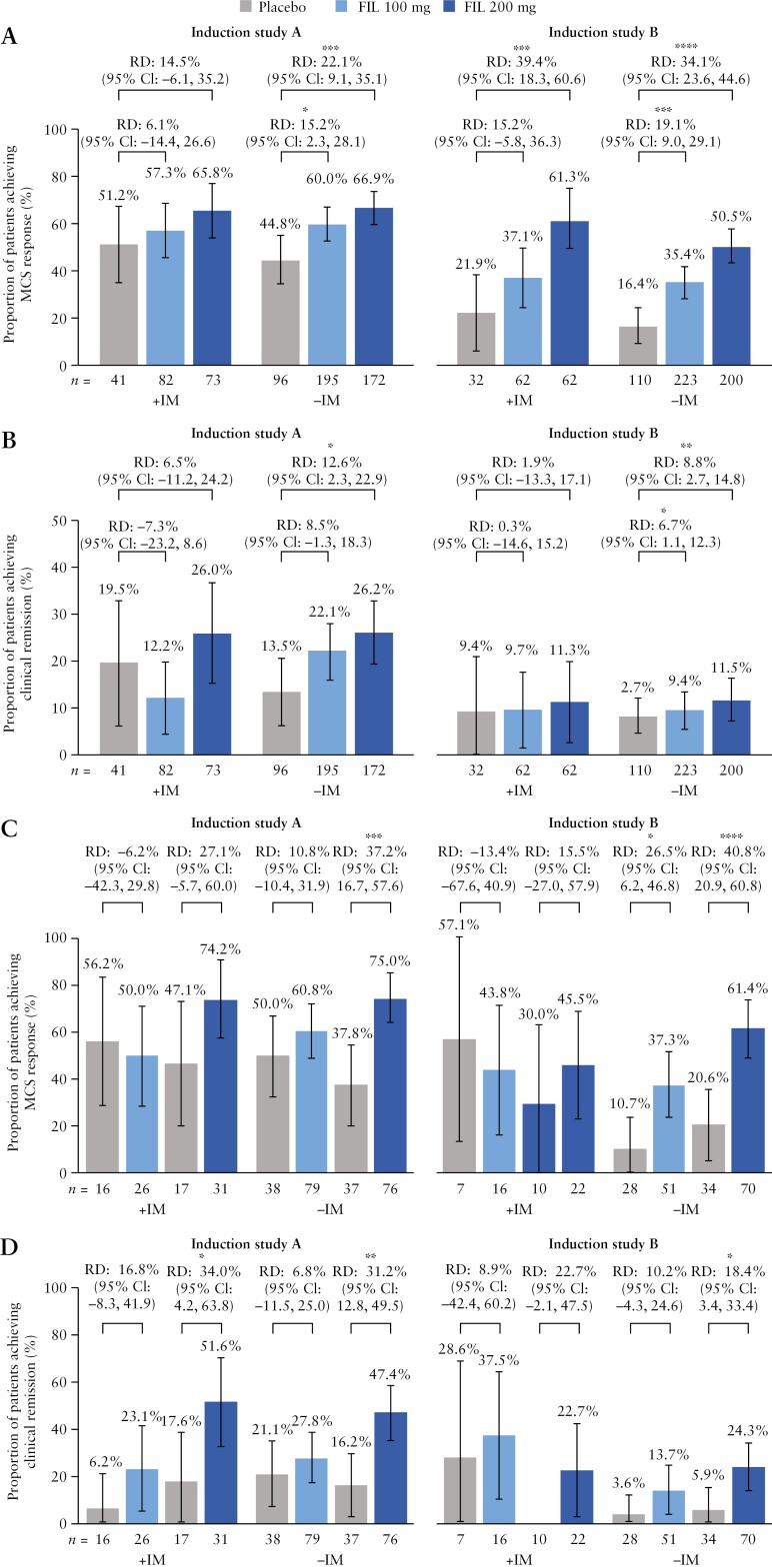
Week 10 MCS response [A] and clinical remission [B], and Week 58 MCS response [C] and clinical remission [D], in patients treated with filgotinib 200 mg or filgotinib 100 mg with or without concomitant IM. CI, confidence interval; FIL, filgotinib; IM, immunomodulator; MCS, Mayo Clinic Score; RD, risk difference. Data shown indicate non-stratified RDs vs placebos [95% CIs]. Error bars indicate 95% CIs. *p < 0.05, **p < 0.01, ***p < 0.001, ****p < 0.0001 vs placebo.

**Figure 2:**
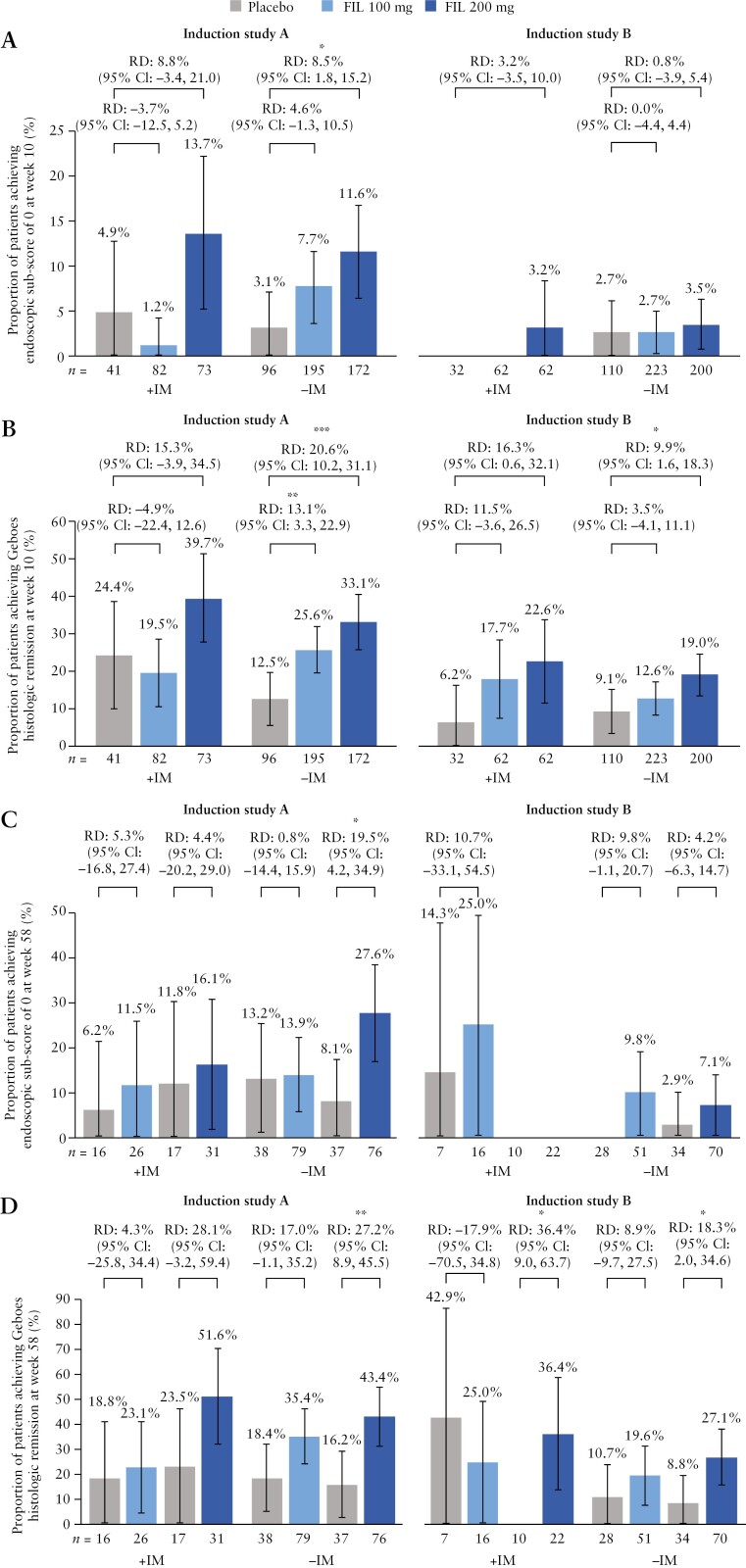
Week 10 endoscopic remission [A] and Geboes histological remission [B], and Week 58 endoscopic remission [C] and Geboes histological remission [D], in patients treated with filgotinib 200 mg or 100 mg with or without concomitant IM. CI, confidence interval; FIL, filgotinib; IM, immunomodulator; RD, risk difference. Data shown indicate non-stratified RDs vs placebos [95% CIs]. Error bars indicate 95% CIs. *p < 0.05, **p < 0.01, ***p< 0.001 vs placebo.

Week 10 response/remission rates were generally lower in the −IM groups than in the +IM groups when considering the placebo arm. Statistically significant RDs in favour of filgotinib 200 mg vs placebo for Week 10 efficacy outcomes were thus more frequent in the −IM groups than in the +IM groups (Figures 1 and 2 [A and B]), particularly in induction study A [biologic-naive patients].

Efficacy outcomes at Week 10 in patients treated with filgotinib 200 mg did not differ between +IM and −IM groups in both biologic-naive and biologic-experienced patients (Figures 1 and 2 [A and B]). In induction study A [biologic-naive patients], similar proportions of patients in the +IM and −IM groups achieved MCS response (65.8% [48/73] vs 66.9% [115/172]), clinical remission (26.0% [19/73] vs 26.2% [45/172]), endoscopic remission (13.7% [10/73] vs 11.6% [20/172]), and Geboes histological remission (39.7% [29/73] vs 33.1% [57/172]). In induction study B [biologic-experienced patients], similar proportions of patients in the +IM and −IM groups achieved MCS response (61.3% [38/62] vs 50.5% [101/200]), clinical remission (11.3% [7/62] vs 11.5% [23/200]), endoscopic remission (3.2% [2/62] vs 3.5% [7/200]), and Geboes histological remission (22.6% [14/62] vs 19.0% [38/200]).

Efficacy outcomes at Week 10 in patients treated with filgotinib 100 mg also did not differ between +IM and −IM groups in both biologic-naive and biologic-experienced patients (Figures 1 and 2 [A and B]). In induction study A [biologic-naive patients], similar proportions of patients in the +IM and −IM groups achieved MCS response (57.3% [47/82] vs 60.0% [117/195]), clinical remission (12.2% [10/82] vs 22.1% [43/195]), endoscopic remission (1.2% [1/82] vs 7.7% [15/195]), and Geboes histological remission (19.5% [16/82] vs 25.6% [50/195]). In induction study B [biologic-experienced patients], similar proportions of patients in the +IM and −IM groups achieved MCS response (37.1% [23/62] vs 35.4% [79/223]), clinical remission (9.7% [6/62] vs 9.4% [21/223]), endoscopic remission (0% [0/62] vs 2.7% [6/223]), and Geboes histological remission (17.7% [11/62] vs 12.6% [28/223]).

### 3.3. Efficacy outcomes with filgotinib maintenance with or without IM

Similar to induction, Week 58 response/remission rates were generally lower in the −IM groups than in the +IM groups when considering the placebo arm. Statistically significant RDs in favour of filgotinib 200 mg vs placebo for Week 58 efficacy outcomes were thus more frequent in the −IM groups than in the +IM groups (Figures 1 and 2 [C and D]), particularly in biologic-naive patients.

Efficacy outcomes at Week 58 in patients treated with filgotinib 200 mg [following an induction dose of filgotinib 200 mg] did not differ between +IM and −IM groups in both biologic-naive and biologic-experienced patients (Figures 1 and 2 [C and D]). In induction study A [biologic-naive patients], similar proportions of patients in the +IM and −IM groups achieved MCS response (74.2% [23/31] vs 75.0% [57/76]), clinical remission (51.6% [16/31] vs 47.4% [36/76]), endoscopic remission (16.1% [5/31] vs 27.6% [21/76]), and Geboes histological remission (51.6% [16/31] vs 43.4% [33/76]). In induction study B [biologic-experienced patients], similar proportions of patients in the +IM and −IM groups achieved MCS response (45.5% [10/22] vs 61.4% [43/70]), clinical remission (22.7% [5/22] vs 24.3% [17/70]), endoscopic remission (0% [0/22] vs 7.1% [5/70]), and Geboes histological remission (36.4% [8/22] vs 27.1% [19/70]).

Similarly, efficacy outcomes at Week 58 in patients treated with filgotinib 100 mg [following an induction dose of filgotinib 100 mg] did not differ between +IM and −IM groups in both biologic-naive and biologic-experienced patients (Figures 1 and 2 [C and D]). In induction study A [biologic-naive patients], similar proportions of patients in the +IM and −IM groups achieved MCS response (50.0% [13/26] vs 60.8% [48/79]), clinical remission (23.1% [6/26] vs 27.8% [22/79]), endoscopic remission (11.5% [3/26] vs 13.9% [11/79]), and Geboes histological remission (23.1% [6/26] vs 35.4% [28/79]). In induction study B [biologic-experienced], similar proportions of patients in the +IM and −IM groups achieved MCS response (43.8% [7/16] vs 37.3% [19/51]), clinical remission (37.5% [6/16] vs 13.7% [7/51]), endoscopic remission (25.0% [4/16] vs 9.8% [5/51]), and Geboes histological remission (25.0% [4/16] vs 19.6% [10/51]).

### 3.4. Disease worsening with filgotinib maintenance with or without IM

During the maintenance study, a significantly lower probability of disease worsening was observed in the −IM group in patients treated with filgotinib 200 mg vs placebo [HR, 0.23; 95% CI: 0.15, 0.37; *p* = 0.001] and filgotinib 100 mg vs placebo [HR, 0.49; 95% CI: 0.33, 0.72; *p* = 0.001] [Supplementary Table 5]. No differences were observed in the +IM group in patients treated with filgotinib [either 200 mg or 100 mg] vs placebo [Supplementary Table 5].

**Figure 3:**
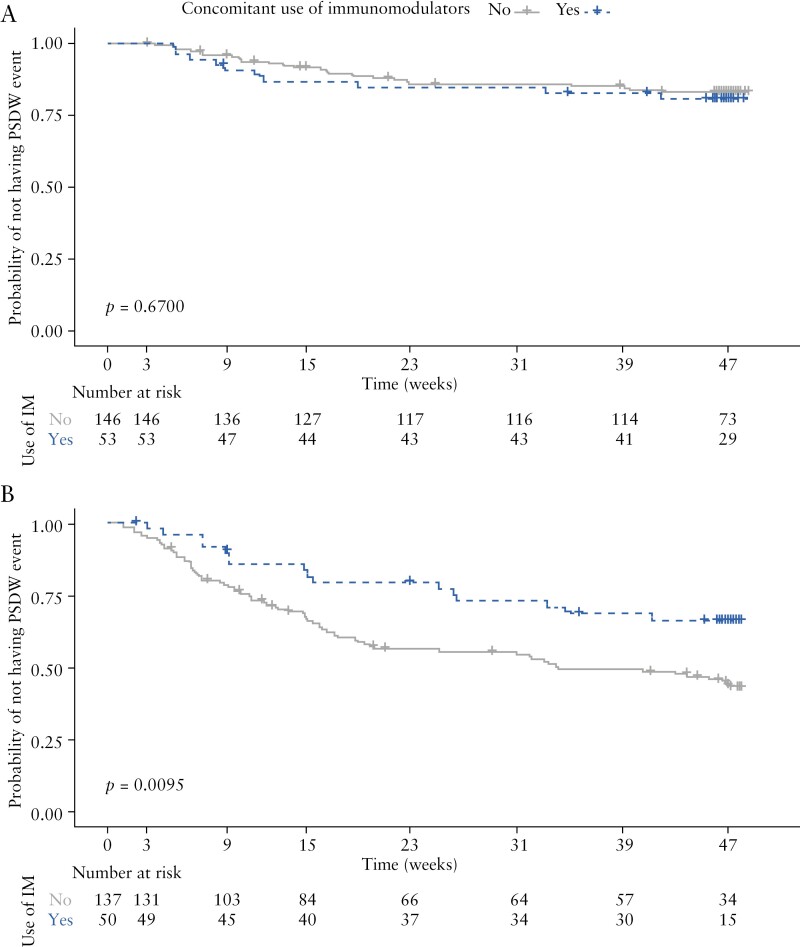
Kaplan–Meier curves for time to PSDW during the maintenance study with and without concomitant IM use in filgotinib 200 mg [A] and all placebo groups combined [B]. IM, immunomodulator; PSWD, protocol-specified disease worsening.

The probability of protocol-specified disease worsening during the maintenance study in patients treated with filgotinib 200 mg did not differ between +IM and −IM groups [*p* = 0.6700] [Figure 3A]. Similar findings were observed in the filgotinib 100 mg group [*p* = 0.7600] [Supplementary Figure 2A] and in the combined filgotinib dose group [*p* = 0.6800] [Supplementary Figure 2B]. In patients receiving placebo, the probability of protocol-specified disease worsening was significantly lower in the +IM group than in the −IM group [*p* = 0.0095] [Figure 3B].

### 3.5. Safety

Tables 2 and 3 show the incidences of treatment-emergent AEs by concomitant IM use at Week 10 [induction studies A and B combined] and Week 58 [maintenance study], respectively. No notable differences were observed in the incidences of any AEs between +IM and −IM groups in the induction or maintenance studies. The proportions of patients with infections and infestations in the filgotinib 200 mg and filgotinib 100 mg induction studies [A and B combined] were 16.3% [22/135] in the +IM group and 18.8% [70/372] in the −IM group, and 12.5% [18/144] in the +IM group and 15.3% [64/418] in the −IM group, respectively. The proportions of patients with any infections and infestations in the filgotinib 200 mg and filgotinib 100 mg maintenance groups [of those who received induction filgotinib 200 mg and induction filgotinib 100 mg, respectively] were 24.1% [13/54] in the +IM group and 39.2% [58/148] in the −IM group, and 31.8% [14/44] in the +IM group and 23.7% [32/135] in the −IM group, respectively. Given that AEs of infection were not specifically categorised as viral vs other types during the SELECTION trial, the proportions of patients with viral infections in this cohort are unknown. The proportions of patients with viral herpes zoster in the filgotinib 200 mg and filgotinib 100 mg induction studies were 0.8% [3/372] and 0.2% [1/418], respectively, in the −IM groups only. The proportions of patients with viral herpes zoster in the filgotinib 200 mg and placebo maintenance groups [of those who received induction filgotinib 200 mg and induction filgotinib 100 mg, respectively] were 1.9% [1/54] and 4.2% [1/24], respectively, in the +IM groups only. Further data on rates of viral herpes zoster infection with filgotinib treatment during SELECTION have recently been published.^20^ Only one AE of opportunistic infection was reported during SELECTION.^8^ Two deaths were reported in patients receiving maintenance filgotinib 200 mg, one each in the +IM and −IM groups; neither was considered related to treatment.

**Table 2.** Summary of treatment-emergent AEs at Week 10 by treatment group and concomitant IM use.

Induction	FIL 200 mg [*N* = 507]		FIL 100 mg [*N* = 562]		PBO [*N* = 279]	
AEs, *n* [%]	+IM^a^ [*n* = 135]	−IM [*n* = 372]	+IM^a^ [*n* = 144]	−IM [*n* = 418]	+IM^a^ [*n* = 73]	−IM [*n* = 206]
Any AE	70 [51.9]	202 [54.3]	73 [50.7]	210 [50.2]	46 [63.0]	111 [53.9]
Any SAE	1 [0.7]	21 [5.6]	8 [5.6]	20 [4.8]	3 [4.1]	10 [4.9]
Any infections and infestation	22 [16.3]	70 [18.8]	18 [12.5]	64 [15.3]	15 [20.5]	24 [11.7]
Any serious infection	0 [0]	3 [0.8]	1 [0.7]	5 [1.2]	1 [1.4]	2 [1.0]
Any herpes zoster infection	0 [0]	3 [0.8]	0 [0]	1 [0.2]	0 [0]	0 [0]
Any pulmonary embolism	0 [0]	1 [0.3]	0 [0]	0 [0]	0 [0]	0 [0]

AE, adverse event; FIL, filgotinib; IM, immunomodulator; PBO, placebo; SAE, serious adverse event.

^a^+IM includes azathioprine, 6-mercaptopurine, or methotrexate.

**Table 3:** Summary of treatment-emergent AEs at Week 58 by treatment group and concomitant IM use.

Induction	FIL 200 mg				FIL 100 mg				PBO	
Maintenance	FIL 200 mg		PBO		FIL 100 mg		PBO		PBO	
AEs, *n* [%]	+IM^a^ [*n *= 54]	−IM [*n* = 148]	+IM^a^ [*n* = 27]	−IM [*n* *=* 72]	+IM^a^ [*n* = 44]	−IM [*n* = 135]	+IM^a^ [*n* = 24]	−IM [*n* = 67]	+IM^a^ [*n* = 30]	−IM [*n* = 63]
Any AE	34 [63.0]	101 [68.2]	20 [74.1]	39 [54.2]	31 [70.5]	77 [57.0]	12 [50.0]	48 [71.6]	22 [73.3]	35 [55.6]
Any SAE	4 [7.4]	5 [3.4]	0 [0]	0 [0]	2 [4.5]	6 [4.4]	1 [4.2]	6 [9.0]	2 [6.7]	2 [3.2]
Any infection and infestation	13 [24.1]	58 [39.2]	9 [33.3]	16 [22.2]	14 [31.8]	32 [23.7]	5 [20.8]	22 [32.8]	11 [36.7]	10 [15.9]
Any serious infection	1 [1.9]	1 [0.7]	0 [0]	0 [0]	1 [2.3]	2 [1.5]	0 [0]	2 [3.0]	0 [0]	1 [1.6]
Any herpes zoster infection	1 [1.9]	0 [0]	0 [0]	0 [0]	0 [0]	0 [0]	1 [4.2]	0 [0]	0 [0]	0 [0]
Any pulmonary embolism	0 [0]	0 [0]	0 [0]	0 [0]	0 [0]	0 [0]	0 [0]	0 [0]	0 [0]	0 [0]
Any AE leading to death	1 [1.9]	1 [0.7]	0 [0]	0 [0]	0 [0]	0 [0]	0 [0]	0 [0]	0 [0]	0 [0]

AE, adverse event; FIL, filgotinib; IM, immunomodulator; PBO, placebo; SAE, serious adverse event.

^a^+IM includes azathioprine, 6-mercaptopurine, or methotrexate.

Although not included among our initial planned analyses, leukopenia AE numbers by IM use are also provided in Supplementary Table 6 [induction study] and Supplementary Table 7 [maintenance study]. Similarly, rates of AEs, SAEs, and infection and infestation AEs by concomitant IM and corticosteroid use are provided in Supplementary Table 8 [induction study]. In general, the rates of infection and infestation AEs tended to be numerically higher in patients with concomitant corticosteroid use than without, irrespective of concomitant IM use or dosage of filgotinib; however, no clear signals were observed in terms of differences in AE or SAE incidences between groups. The main purpose of including baseline corticosteroid use data in Table 1 was to demonstrate that this was not a confounding variable [ie, rates of corticosteroid use were similar between +IM and −IM groups]. We have not included data by concomitant corticosteroid use for the maintenance study in the manuscript because patients were actively tapered off steroid therapy ⁓4 weeks into the 48-week maintenance phase. The data on the impact of concomitant corticosteroids on safety have recently been reported for the SELECTION study.^21^

## 4. Discussion

In these post hoc analyses, we assessed the impact of concomitant IM use on the efficacy and safety of filgotinib treatment in patients with moderately to severely active UC from the phase 2b/3 SELECTION study. During both the 10-week induction and 47-week maintenance phases, the proportions of patients achieving clinical remission, MCS response, endoscopic remission, and Geboes histological remission, or with treatment-emergent AEs, were similar with or without concomitant IM use in biologic-naive and biologic-experienced patients. Consistent with these findings, the probability of protocol-specified disease worsening during maintenance filgotinib [200 mg or 100 mg] treatment was not significantly different in patients receiving a concomitant IM and those not receiving a concomitant IM. Overall, our results suggest that the efficacy and safety profiles of filgotinib 200 mg treatment are similar with or without concomitant IM use.

Our data indicate that IM use alone is beneficial for the treatment of UC, because the probability of protocol-specified disease worsening was lower in patients receiving placebo with a concomitant IM than in those without a concomitant IM. Consistent with these findings, placebo-response/remission rates for efficacy outcomes were generally higher in patients who received concomitant IM than in those who did not. This probably contributed to smaller [and less often statistically significant] differences between filgotinib and placebo in patients receiving concomitant IM vs those not receiving concomitant IM, although numerical trends favouring filgotinib were still maintained.

Of note, there were no obvious additive or synergistic effects with the combination of filgotinib and IM in our study, whereas infliximab and AZA combination therapy improved corticosteroid-free remission rates compared with either agent alone, and concomitant IM use with vedolizumab has been previously shown to have some synergistic benefit.^9,15^ IMs, such as MP and AZA, are thought to act by inhibiting lymphocyte proliferation via the incorporation of active drug metabolites into cellular nucleotides, which likely results in anti-inflammatory effects through the suppression of T-cell function and natural killer cell activity.^22^ JAK inhibitors also suppress T-cell/natural killer cell function by suppressing the signalling of multiple cytokines. Therefore, the mode of action of IMs is thought to overlap with that of JAK inhibitors and could explain why we observed no obvious additive or synergistic effects with the combination of filgotinib and IM.

Other JAK inhibitors indicated for the treatment of moderately to severely active UC include tofacitinib and upadacitinib.^16,17^ To our knowledge, however, filgotinib is the only JAK inhibitor with efficacy and safety data by concomitant IM use from a randomised, controlled, clinical trial [the SELECTION study]. In the OCTAVE trials investigating tofacitinib induction and maintenance therapy in patients with moderately to severely active UC, concomitant use of AZA, MP, and methotrexate was prohibited.^23^ In the U-ACHIEVE induction, U-ACCOMPLISH, and U-ACHIEVE maintenance trials evaluating upadacitinib for induction and maintenance therapy, patients receiving AZA or MP 10 days before baseline were excluded.^24^ As a result, treatment with tofacitinib or upadacitinib in combination with potent immunosuppressants, such as AZA, is not recommended for patients with moderate to severe UC.^16,17^ Based on the acceptable safety profile reported in the present study, concomitant use of an IM with filgotinib appears feasible.

An interesting observation with regard to the SELECTION trial relates to the unusually high rate of histological remission [35.1%] achieved with filgotinib 200 mg treatment relative to endoscopic [12.2%] and even clinical [26.1%] remission rates.^8^ These findings are mirrored in the current post hoc analysis of SELECTION trial data by concomitant IM use. Higher histological vs endoscopic remission has also been observed in at least one other trial: a phase 2b investigation of the JAK inhibitor upadacitinib for the treatment of UC.^25^ A reason for these observations could be that endoscopic assessments measure the degree of healing and associated clinical signs at a macroscopic level, which potentially come after [and as a result of] histological signs of healing at the microscopic level. Consistent with this, rates of histological remission in SELECTION were better aligned with endoscopic response [endoscopic score of 0 or 1]. A detailed assessment is beyond the scope of the current analysis, and a future exploration to address this topic is required.

In the present study, the safety profile of filgotinib was similar with and without concomitant IM use over the 58 weeks of the SELECTION study. No notable differences in the proportions of patients experiencing treatment-emergent AEs were observed between patients with or without treatment with a concomitant IM. Based on results from the present study, there is no evidence to suggest that concomitant use of IMs with filgotinib increases the risk of safety concerns. Complementing these findings, data from the multicentre, double-blind, randomised, controlled, phase 3 FINCH 3 trial in patients with active rheumatoid arthritis found that the number of treatment-emergent AEs over 52 weeks were similar in filgotinib 200 mg plus methotrexate, filgotinib 100 mg plus methotrexate, filgotinib 200 mg only, and methotrexate only groups.^26^ In the FINCH 3 trial, filgotinib in combination with methotrexate also led to significant improvements in rheumatoid arthritis symptoms compared with methotrexate alone in patients with limited or without previous methotrexate treatment.^26^ Moreover, in the phase 3b/4 ORAL Strategy trial, tofacitinib monotherapy was not declared non-inferior to the concomitant use of methotrexate and tofacitinib in patients with rheumatoid arthritis.^27^

A key strength of our study is that it is the first to evaluate the efficacy and safety of concomitant IM use with a JAK inhibitor in patients with UC. Limitations of the current study include the post hoc nature of the analyses, and the fact that thiopurine methyltransferase [*TPMT*] and nudix hydrolase 15 [*NUDT15*] genotypes, as well as 6-thioguanine nucleotide levels, were not assessed, which could have population-based implications for the efficacy and safety of concomitant IM use with filgotinib. In addition, because no IM dosing guidance was given during the maintenance study, adjustments may have occurred during this period. It is also theoretically possible that some IM use may have been incidental to UC [eg, if a patient was eligible to enter the study based on corticosteroid-refractory disease, but also happened to be taking an IM for a comorbid inflammatory condition]. However, given the study population, such instances [if any] are likely to be rare, and the effects on our analysis extremely minimal. Studies of longer duration and/or those with optimised IM use are required to show the possible benefits of concomitant IM use with filgotinib.

In conclusion, the efficacy and safety profiles of filgotinib treatment in the SELECTION study did not differ with or without concomitant IM [predominantly AZA] treatment. Additional clinical studies, and prospective, longer-term, real-world data are required to further assess and confirm the efficacy and safety profiles of filgotinib treatment with concomitant IM use.

## Supplementary Data

Supplementary data are available at *ECCO-JCC* online.

jjad201_suppl_Supplementary_Figures_S1-S2_Tables_S1-S8

## Data Availability

Gilead Sciences shares anonymised individual patient data upon request or as required by law or regulation with qualified external researchers, based on submitted curriculum vitae and reflecting non conflict of interest. The request proposal must also include a statistician. Approval of such requests is at Gilead Science’s discretion and is dependent on the nature of the request, the merit of the research proposed, the availability of the data, and the intended use of the data. Data requests should be sent to datarequest@gilead.com.
