# Supplementary material for: Impact of Concomitant Thiopurine on the Efficacy and Safety of Filgotinib in Patients with Ulcerative Colitis: Post Hoc Analysis of the Phase 2b/3 SELECTION Study
Source: J Crohns Colitis. 2023 Nov 29;18(6):801–11. doi: 10.1093/ecco-jcc/jjad201 (PMC11147794; doi:10.1093/ecco-jcc/jjad201)
Supplement: jjad201_suppl_Supplementary_Figures_S1-S2_Tables_S1-S8 [file jjad201_suppl_supplementary_figures_s1-s2_tables_s1-s8.docx]

# Supplementary tables and figures

**Supplementary Table 1.** Baseline patient characteristics in induction studies A and B by type of concomitant IM use.

|  | **Induction study A: biologic-naive patients** | | | | | | | | | | | | **Induction study B: biologic-experienced patients** | | | | | | | | | | | |
| --- | --- | --- | --- | --- | --- | --- | --- | --- | --- | --- | --- | --- | --- | --- | --- | --- | --- | --- | --- | --- | --- | --- | --- | --- |
|  | **FIL 200 mg** | | | | **FIL 100 mg** | | | | **PBO** | | | | **FIL 200 mg** | | | | **FIL 100 mg** | | | | **PBO** | | | |
|  | +AZA  *n* = 61 | +IM^a^  *n* = 73 | −IM  *n* = 172 | Total  *N* = 245 | +AZA  *n* = 73 | +IM^a^  *n* = 82 | −IM  *n* = 195 | Total  *N* = 277 | +AZA  *n* = 39 | +IM^a^  *n* = 41 | −IM  *n* = 96 | Total  *N* = 137 | +AZA  *n* = 44 | +IM^a^  *n* = 62 | −IM  *n* = 200 | Total  *N* = 262 | +AZA  *n* = 47 | +IM^a^  *n* = 62 | −IM  *n* = 223 | Total  *N* = 285 | +AZA  *n* = 24 | +IM^a^  *n* = 32 | −IM  *n* = 110 | Total  *N* = 142 |
| Age, years, mean (SD) | 40.9 (11.77) | 42.2 (12.56) | 42.4 (13.33) | 42.3 (13.08) | 42.3 (12.70) | 42.6 (12.40) | 42.2 (13.62) | 42.3 (13.25) | 40.5 (11.55) | 40.7 (11.54) | 41.5 (13.43) | 41.3 (12.86) | 40.4 (15.16) | 40.8 (15.56) | 44.0 (13.67) | 43.3 (14.17) | 39.2 (13.01) | 40.6  (13.27) | 43.6 (14.48) | 42.9 (14.25) | 36.8 (14.17) | 39.2 (15.01) | 45.9 (14.62) | 44.4 (14.92) |
| Female sex, *n* (%) | 27 (44.3) | 31 (42.5) | 91 (52.9) | 122 (49.8) | 34 (46.6) | 38 (46.3) | 82 (42.1) | 120 (43.3) | 12 (30.8) | 14 (34.1) | 36 (37.5) | 50 (36.5) | 20 (45.5) | 29 (46.8) | 85 (42.5) | 114 (43.5) | 13 (27.7) | 18  (29.0) | 81 (36.3) | 99 (34.7) | 8 (33.3) | 10 (31.2) | 46 (41.8) | 56 (39.4) |
| Body weight, median (Q1–Q3) | 66.2  (57.20–83.00) | 69.2 (57.60–84.00) | 66.0 (56.00–79.15) | 66.2 (57.00–81.00) | 63.2 (52.90–73.80) | 62.8 (52.45–74.85) | 68.5 (58.20–82.55) | 66.8 (56.90–81.00) | 62.0 (57.70–77.50) | 62.0 (57.00–79.00) | 68.0 (58.00–81.20) | 66.5 (58.00–80.50) | 66.5 (61.03–77.28) | 66.0 (57.28–76.62) | 72.0 (61.00–86.62) | 70.9 (60.42–84.07) | 70.5 (63.75–79.50) | 71.0 (60.47-81.90) | 73.4 (63.00–85.05) | 72.5 (62.50–84.00) | 71.0 (62.85–84.00) | 71.0 (61.40–85.90) | 71.6 (59.70–84.15) | 71.2 (60.12–84.88) |
| BMI, median (Q1–Q3) | 23.9 (20.41–27.80) | 24.1 (21.13–27.80) | 23.9 (20.41–27.94) | 23.9 (20.41–27.92) | 22.5 (19.98–26.02) | 22.9 (19.98–26.04) | 24.0 (21.19–27.88) | 23.6 (20.60–27.41) | 22.8 (20.40–26.39) | 22.9 (20.42–26.58) | 23.8 (20.83–26.85) | 23.2 (20.76–26.85) | 23.9 (21.10–27.36) | 23.4 (20.76–27.17) | 24.4 (21.50–28.66) | 24.3 (21.21–27.91) | 23.1 (20.63–25.83) | 23.9 (20.68-26.33) | 24.7 (22.04–27.87) | 24.4 (21.93–27.54) | 22.9 (20.53–25.82) | 23.1 (20.53–28.32) | 24.5 (21.14–27.75) | 24.0 (21.08–28.15) |
| Smoking status, *n* (%) |  |  |  |  |  |  |  |  |  |  |  |  |  |  |  |  |  |  |  |  |  |  |  |  |
| Current | 3 (4.9) | 3 (4.1) | 12 (7) | 15 (6.1) | 5 (6.8) | 5 (6.1) | 5 (2.6) | 10 (3.6) | 1 (2.6) | 1 (2.4) | 4 (4.2) | 5 (3.6) | 4 (9.1) | 4 (6.5) | 4 (2.0) | 8 (3.1) | 1 (2.1) | 2 (3.2) | 19 (8.5) | 21 (7.4) | 0 | 0 | 5 (4.5) | 5 (3.5) |
| Former | 16 (26.2) | 22 (30.1) | 33 (19.2) | 55 (22.4) | 14 (19.2) | 18 (22.0) | 36 (18.5) | 54 (19.5) | 6 (15.4) | 6 (14.6) | 16 (16.7) | 22 (16.1) | 6 (13.6) | 11 (17.7) | 62 (31) | 73 (27.9) | 11 (23.4) | 17 (27.4) | 76 (34.1) | 93 (32.6) | 3 (12.5) | 8  (25.0) | 35 (31.8) | 43 (30.3) |
| Never | 42  (68.9) | 48 (65.8) | 127 (73.8) | 175 (71.4) | 54 (74.0) | 59 (72.0) | 154 (79) | 213 (76.9) | 32 (82.1) | 34 (82.9) | 76 (79.2) | 110 (80.3) | 34 (77.3) | 47 (75.8) | 134 (67.0) | 181 (69.1) | 35 (74.5) | 43  (69.4) | 128 (57.4) | 171 (60.0) | 21 (87.5) | 24 (75.0) | 70 (63.6) | 94 (66.2) |
| Duration of UC, years, mean (SD) | 7.0  (5.29) | 6.8 (5.42) | 7.3 (7.41) | 7.2 (6.87) | 6.5 (7.24) | 7.1 (8.46) | 6.5 (6.94) | 6.7 (7.41) | 5.4 (4.27) | 5.5 (4.18) | 6.8 (8.39) | 6.4 (7.39) | 8.9 (7.20) | 8.7 (6.67) | 10.2 (7.90) | 9.8 (7.64) | 8.5 (7.11) | 8.1  (6.70) | 10.2 (7.23) | 9.7 (7.15) | 8.1 (7.22) | 9.3 (7.55) | 10.4 (8.42) | 10.2 (8.22) |
| Total Mayo Clinic Score, mean (SD) | 8.4  (1.30) | 8.4 (1.23) | 8.7 (1.33) | 8.6 (1.31) | 8.7 (1.34) | 8.7 (1.36) | 8.5 (1.45) | 8.6 (1.43) | 8.7 (1.28) | 8.7 (1.26) | 8.7 (1.35) | 8.7 (1.32) | 9.4 (1.57) | 9.2 (1.61) | 9.2 (1.32) | 9.2 (1.39) | 9.3 (1.37) | 9.3  (1.26) | 9.3 (1.28) | 9.3 (1.27) | 9.0 (1.44) | 9.1 (1.39) | 9.3 (1.43) | 9.3 (1.42) |
| Mayo endoscopic score of 3, *n* (%) | 38  (62.3) | 45 (61.6) | 88 (51.2) | 133 (54.3) | 50 (68.5) | 56 (68.3) | 103 (52.8) | 159 (57.4) | 25 (64.1) | 25 (61) | 51 (53.1) | 76 (55.5) | 38 (86.4) | 53 (85.5) | 150 (75.0) | 203 (77.5) | 39 (83.0) | 51  (82.3) | 171 (76.7) | 222 (77.9) | 20 (83.3) | 26 (81.2) | 85 (77.3) | 111 (78.2) |
| C-reactive protein, mg/L, mean (SD) | 11.9 (26.31) | 11.6 (24.58) | 7.4 (10.88) | 8.6 (16.27) | 9.4 (23.78) | 8.8 (22.55) | 7.3 (14.73) | 7.7 (17.38) | 4.6 (6.88) | 4.6 (6.73) | 6.3 (7.92) | 5.8 (7.60) | 10.1 (11.85) | 10.2 (11.98) | 12.8 (15.61) | 12.2 (14.85) | 6.3 (8.61) | 8.9  (15.30) | 12.5 (18.62) | 11.7 (17.99) | 7.4 (9.70) | 7.4 (8.85) | 15.9 (26.91) | 14.0 (24.28) |
| Faecal calprotectin, μg/g, mean (SD) | 1716.4 (2219.39) | 1612.8 (2074.37) | 2246.0 (2827.68) | 2058.7 (2639.06) | 2184.9 (4142.73) | 2041.5 (3934.12) | 1983.2 (3230.70) | 2000.6 (3447.81) | 2571.0 (3785.44) | 2473.7 (3715.21) | 2125.8 (2507.12) | 2231.5 (2916.92) | 2344.0 (4256.73) | 2070.8 (3662.60) | 3078.7 (4174.15) | 2844.6 (4076.46) | 2143.7 (3339.52) | 2035.0 (3094.51) | 2292.8 (3099.84) | 2236.2 (3094.93) | 1417.8 (1269.24) | 1470.5 (1264.54) | 2756.1 (3938.10) | 2478.7 (3571.41) |
| Concomitant use of systemic corticosteroids only, *n* (%) | 0 | 0 | 54 (31.4) | 54 (22.0) | 0 | 0 | 67 (34.4) | 67 (24.2) | 0 | 0 | 34 (35.4) | 34 (24.8) | 0 | 0 | 94 (47.0) | 94 (35.9) | 0 | 0 | 103 (46.2) | 103 (36.1) | 0 | 0 | 51 (46.4) | 51 (35.9) |
| Concomitant use of IMs only, *n* (%) | 42 (68.9) | 53 (72.6) | 0 | 53 (21.6) | 56 (76.7) | 63 (76.8) | 0 | 63 (22.7) | 31 (79.5) | 33 (80.5) | 0 | 33 (24.1) | 24 (54.5) | 34 (54.8) | 0 | 34 (13.0) | 27 (57.4) | 34 (54.8) | 0 | 34 (11.9) | 14 (58.3) | 21 (65.6) | 0 | 21 (14.8) |
| Concomitant use of systemic corticosteroids and IMs, *n* (%) | 19 (31.1) | 20 (27.4) | 0 | 20 (8.2) | 17 (23.3) | 19 (23.2) | 0 | 19 (6.9) | 8 (20.5) | 8 (19.5) | 0 | 8 (5.8) | 20 (45.5) | 28 (45.2) | 0 | 28 (10.7) | 20 (42.6) | 28 (45.2) | 0 | 28 (9.8) | 10 (41.7) | 11 (34.4) | 0 | 11 (7.7) |
| Prednisone-equivalent dose (mg/day), median (Q1–Q3) | 20.0 (10.00-30.00) | 17.5 (10.00-30.00) | 20.0 (10.00-25.00) | 20.0 (10.00-25.00) | 15.0 (7.50-20.00) | 15.0 (8.75-20.00) | 20.0 (10.00-25.83) | 15.0 (10.00-25.00) | 20.0 (10.00-22.50) | 20.0 (10.00-22.50) | 20.0 (15.00-30.00) | 20.0 (15.00-30.00) | 17.5 (10.00-21.25) | 15.0 (10.00-20.00) | 15.0 (10.00-20.00) | 15.0 (10.00-20.00) | 20.0 (8.75-20.00) | 20.0 (8.75-20.00) | 20.0 (10.00-20.00) | 20.0 (10.00-20.00) | 13.8 (6.25-20.00) | 15.0 (7.50-20.00) | 20.0 (10.00-20.00) | 20.0 (10.00-20.00) |
| Concomitant use of 5-ASA, *n* (%) | 59 (96.7) | 68 (93.2) | 151 (87.8) | 219 (89.4) | 62 (84.9) | 68 (82.9) | 177 (90.8) | 245 (88.4) | 33 (84.6) | 35 (85.4) | 83 (86.5) | 118 (86.1) | 31 (70.5) | 45 (72.6) | 108 (54.0) | 153 (58.4) | 41 (87.2) | 48  (77.4) | 119 (53.4) | 167 (58.6) | 18 (75.0) | 23 (71.9) | 60 (54.5) | 83 (58.5) |
| Number of prior biologic agents used, *n* (%) |  |  |  |  |  |  |  |  |  |  |  |  |  |  |  |  |  |  |  |  |  |  |  |  |
| 0 | 61 (100.0) | 73 (100.0) | 172 (100.0) | 245 (100.0) | 72 (98.6) | 81 (98.8) | 194 (99.5) | 275 (99.3) | 39 (100.0) | 41 (100.0) | 96 (100.0) | 137 (100.0) | 0 | 0 | 3  (1.5) | 3  (1.1) | 1  (2.1) | 1  (1.6) | 1  (0.4) | 2  (0.7) | 0 | 0 | 3  (2.7) | 3  (2.1) |
| 1 | 0 | 0 | 0 | 0 | 1 (1.4) | 1 (1.2) | 0 | 1 (0.4) | 0 | 0 | 0 | 0 | 18 (40.9) | 25 (40.3) | 55 (27.5) | 80 (30.5) | 24 (51.1) | 29 (46.8) | 69 (30.9) | 98 (34.4) | 13 (54.2) | 14 (43.8) | 32 (29.1) | 46 (32.4) |
| 2 | 0 | 0 | 0 | 0 | 0 | 0 | 1 (0.5) | 1 (0.4) | 0 | 0 | 0 | 0 | 13 (29.5) | 19 (30.6) | 71 (35.5) | 90 (34.4) | 17 (36.2) | 21 (33.9) | 88 (39.5) | 109 (38.2) | 5 (20.8) | 11 (34.4) | 34 (30.9) | 45 (31.7) |
| 3 | 0 | 0 | 0 | 0 | 0 | 0 | 0 | 0 | 0 | 0 | 0 | 0 | 13 (29.5) | 18 (29.0) | 71 (35.5) | 89 (34.0) | 5 (10.6) | 11 (17.7) | 65 (29.1) | 76 (26.7) | 6 (25.0) | 7 (21.9) | 41 (37.3) | 48 (33.8) |
| Prior use of at least 1 TNF antagonist, *n* (%) | 0 | 0 | 0 | 0 | 1 (1.4) | 1 (1.2) | 1 (0.5) | 2 (0.7) | 0 | 0 | 0 | 0 | 42 (95.5) | 59 (95.2) | 183 (91.5) | 242 (92.4) | 43 (91.5) | 57 (91.9) | 209 (93.7) | 266 (93.3) | 22 (91.7) | 30 (93.8) | 100 (90.9) | 130 (91.5) |
| Prior use of vedolizumab, *n* (%) | 0 | 0 | 0 | 0 | 0 | 0 | 1 (0.5) | 1 (0.4) | 0 | 0 | 0 | 0 | 22 (50.0) | 29 (46.8) | 135 (67.5) | 164 (62.6) | 15 (31.9) | 26 (41.9) | 119 (53.4) | 145 (50.9) | 11 (45.8) | 17 (53.1) | 68 (61.8) | 85 (59.9) |
| Prior use of at least 1 TNF antagonist and vedolizumab, *n* (%) | 0 | 0 | 0 | 0 | 0 | 0 | 1 (0.5) | 1 (0.4) | 0 | 0 | 0 | 0 | 20 (45.5) | 26 (41.9) | 121 (60.5) | 147 (56.1) | 12 (25.5) | 22 (35.5) | 106 (47.5) | 128 (44.9) | 9 (37.5) | 15 (46.9) | 61 (55.5) | 76 (53.5) |
| Prior failure of at least 1 TNF antagonist, *n* (%) | 0 | 0 | 0 | 0 | 0 | 0 | 1 (0.5) | 1 (0.4) | 0 | 0 | 0 | 0 | 34 (77.3) | 50 (80.6) | 168 (84.0) | 218 (83.2) | 43 (91.5) | 55 (88.7) | 196 (87.9) | 251 (88.1) | 20 (83.3) | 26 (81.2) | 94 (85.5) | 120 (84.5) |
| Prior failure of vedolizumab, *n* (%) | 0 | 0 | 0 | 0 | 0 | 0 | 1 (0.5) | 1 (0.4) | 0 | 0 | 0 | 0 | 20 (45.5) | 27 (43.5) | 121 (60.5) | 148 (56.5) | 14 (29.8) | 24 (38.7) | 108 (48.4) | 132 (46.3) | 10 (41.7) | 16 (50.0) | 60 (54.5) | 76 (53.5) |

5-ASA, 5-aminosalicylic acid; BMI, body mass index; FIL, filgotinib; IM, immunomodulator; PBO, placebo; Q, quartile; SD, standard deviation; TNF, tumour necrosis factor; UC, ulcerative colitis.

^a^+IM includes azathioprine, 6-mercaptopurine, or methotrexate.

**Supplementary Table 2.** Baseline patient characteristics in the maintenance study by concomitant IM use.

| **Induction** | **FIL 200 mg** | | | | | | **FIL 100 mg** | | | | | |
| --- | --- | --- | --- | --- | --- | --- | --- | --- | --- | --- | --- | --- |
| **Maintenance** | **FIL 200 mg** | | | **PBO** | | | **FIL 100 mg** | | | **PBO** | | |
| **Induction study A: biologic-naive** | | | | | | | | | | | | |
|  | +IM^a^  *n* = 31 | −IM  *n* = 76 | Total  *N* = 107 | +IM^a^  *n* = 17 | −IM  *n* = 37 | Total  *N* = 54 | +IM^a^  *n* = 26 | −IM  *n* = 79 | Total  *N* = 105 | +IM^a^  *n* = 16 | −IM  *n* = 38 | Total  *N* = 54 |
| Age, years, mean (SD) | 44.6 (12.58) | 39.7 (13.45) | 41.1 (13.34) | 38.4 (12.58) | 43.4 (13.13) | 41.8 (13.06) | 39.3 (8.85) | 41.7 (12.13) | 41.1 (11.41) | 41.7 (11.82) | 44.0 (15.46) | 43.3 (14.41) |
| Female sex, *n* (%) | 12 (38.7) | 45 (59.2) | 57 (53.3) | 8 (47.1) | 21 (56.8) | 29 (53.7) | 10 (38.5) | 40 (50.6) | 50 (47.6) | 7 (43.8) | 16 (42.1) | 23 (42.6) |
| Body weight, median (Q1–Q3) | 69.9 (58.05–84.75) | 63.5 (55.38–74.78) | 64.2 (55.55–79.00) | 66.2 (55.40–90.00) | 71.4 (55.00–79.90) | 68.8 (55.10–84.83) | 62.8 (51.33–73.60) | 65.3 (55.70–83.55) | 65.0 (54.20–81.10) | 61.9 (55.58–79.93) | 75.0 (58.70–81.73) | 71.7 (58.07–81.15) |
| BMI, median (Q1–Q3) | 24.4 (19.66–29.68) | 22.1 (19.67–25.50) | 22.7 (19.62–26.48) | 24.3 (22.48–27.78) | 26.1 (20.52–29.45) | 25.7 (21.19–28.55) | 23.3 (20.02–25.86) | 23.4 (20.51–28.03) | 23.4 (20.12–27.30) | 21.8 (19.72–25.73) | 23.9 (21.34–28.29) | 23.2 (20.22–27.39) |
| Smoking status, *n* (%) |  |  |  |  |  |  |  |  |  |  |  |  |
| Current | 1 (3.2) | 8 (10.5) | 9 (8.4) | 0 | 0 | 0 | 2 (7.7) | 2 (2.5) | 4 (3.8) | 0 | 1 (2.6) | 1 (1.9) |
| Former | 9 (29.0) | 13 (17.1) | 22 (20.6) | 4 (23.5) | 9 (24.3) | 13 (24.1) | 7 (26.9) | 15 (19.0) | 22 (21.0) | 4 (25.0) | 6 (15.8) | 10 (18.5) |
| Never | 21 (67.7) | 55 (72.4) | 76 (71.0) | 13 (76.5) | 28 (75.7) | 41 (75.9) | 17 (65.4) | 62 (78.5) | 79 (75.2) | 12 (75.0) | 31 (81.6) | 43 (79.6) |
| Duration of UC, years, mean (SD) | 6.9 (6.24) | 7.1 (7.53) | 7.0 (7.15) | 6.4 (4.64) | 9.1 (7.92) | 8.2 (7.12) | 7.6 (10.50) | 7.7 (7.72) | 7.7 (8.43) | 8.7 (10.41) | 4.9 (4.68) | 6.0 (7.01) |
| Total Mayo Clinic Score, mean (SD) | 8.5 (1.29) | 8.8 (1.26) | 8.7 (1.27) | 8.3 (1.21) | 8.8 (1.31) | 8.6 (1.29) | 9.2 (1.35) | 8.8 (1.37) | 8.9 (1.37) | 8.9 (1.20) | 8.7 (1.44) | 8.7 (1.37) |
| Mayo endoscopic score of 3, *n* (%) | 18 (58.1) | 35 (46.1) | 53 (49.5) | 9 (52.9) | 20 (54.1) | 29 (53.7) | 18 (69.2) | 43 (54.4) | 61 (58.1) | 10 (62.5) | 19 (50.0) | 29 (53.7) |
| C-reactive protein, mg/L, mean (SD) | 13.1 (34.82) | 6.6 (9.41) | 8.5 (20.36) | 3.8 (1.98) | 8.0 (13.91) | 6.7 (11.68) | 3.4 (3.70) | 6.0 (12.21) | 5.4 (10.79) | 10.9 (21.88) | 5.6 (5.97) | 7.2 (12.90) |
| Faecal calprotectin, μg/g, mean (SD) | 1668.1 (2185.93) | 2611.7 (3015.81) | 2342.1 (2826.20) | 1481.6 (2530.96) | 2040.1 (2674.48) | 1864.2 (2619.33) | 1373.5 (1696.85) | 1731.2 (3480.44) | 1641.8 (3127.10) | 4648.8 (7722.76) | 2068.7 (2611.37) | 2833.2 (4801.48) |
| Concomitant use of systemic corticosteroids only, *n* (%) | 0 | 26 (34.2) | 26 (24.3) | 0 | 13 (35.1) | 13 (24.1) | 0 | 29 (36.7) | 29 (27.6) | 0 | 14 (36.8) | 14 (25.9) |
| Concomitant use of IMs only, *n* (%) | 22 (71.0) | 0 | 22 (20.6) | 13 (76.5) | 0 | 13 (24.1) | 18 (69.2) | 0 | 18 (17.1) | 12 (75.0) | 1 (2.6) | 13 (24.1) |
| Concomitant use of systemic corticosteroids and IMs, *n* (%) | 9 (29.0) | 0 | 9 (8.4) | 4 (23.5) | 0 | 4 (7.4) | 8 (30.8) | 0 | 8 (7.6) | 4 (25.0) | 0 | 4 (7.4) |
| Prednisone-equivalent dose (mg/day), median (Q1–Q3) | 20.0 (10.00–30.00) | 20.0 (10.00–20.00) | 20.0 (10.00–22.50) | 30.0 (25.00–30.00) | 25.0 (20.00–30.00) | 25.0 (20.00–30.00) | 10.0 (9.38–12.50) | 20.0 (10.00–30.00) | 20.0 (10.00–30.00) | 12.5 (5.00–20.00) | 17.5 (10.62–20.00) | 17.5 (10.00–20.00) |
| Concomitant use of 5-ASA, *n* (%) | 29 (93.5) | 65 (85.5) | 94 (87.9) | 16 (94.1) | 33 (89.2) | 49 (90.7) | 20 (76.9) | 72 (91.1) | 92 (87.6) | 14 (87.5) | 34 (89.5) | 48 (88.9) |
| Number of prior biologic agents used, *n* (%) |  |  |  |  |  |  |  |  |  |  |  |  |
| 0 | 31 (100.0) | 76 (100.0) | 107 (100.0) | 17 (100.0) | 37 (100.0) | 54 (100.0) | 26 (100.0) | 78 (98.7) | 104 (99.0) | 16 (100.0) | 38 (100.0) | 54 (100.0) |
| 2 | 0 | 0 | 0 | 0 | 0 | 0 | 0 | 1 (1.3) | 1 (1.0) | 0 | 0 | 0 |
| Prior use of at least 1 TNF antagonist, *n* (%) | 0 | 0 | 0 | 0 | 0 | 0 | 0 | 1 (1.3) | 1 (1.0) | 0 | 0 | 0 |
| Prior use of vedolizumab, *n* (%) | 0 | 0 | 0 | 0 | 0 | 0 | 0 | 1 (1.3) | 1 (1.0) | 0 | 0 | 0 |
| Prior use of at least 1 TNF antagonist and vedolizumab, *n* (%) | 0 | 0 | 0 | 0 | 0 | 0 | 0 | 1 (1.3) | 1 (1.0) | 0 | 0 | 0 |
| Prior failure of at least 1 TNF antagonist, *n* (%) | 0 | 0 | 0 | 0 | 0 | 0 | 0 | 1 (1.3) | 1 (1.0) | 0 | 0 | 0 |
| Prior failure of vedolizumab, *n* (%) | 0 | 0 | 0 | 0 | 0 | 0 | 0 | 1 (1.3) | 1 (1.0) | 0 | 0 | 0 |
| **Induction study B: biologic-experienced** | | | | | | | | | | | | |
|  | +IM^a^  *n* = 22 | −IM  *n* = 70 | Total  *N* = 92 | +IM^a^  *n* = 10 | −IM  *n* = 34 | Total  *N* = 44 | +IM^a^  *n* = 16 | −IM  *n* = 51 | Total  *N* = 67 | +IM^a^  *n* = 7 | −IM  *n* = 28 | Total  *N* = 35 |
| Age, years, mean (SD) | 44.5 (17.22) | 45.9 (13.14) | 45.6 (14.13) | 32.3 (9.93) | 46.4 (12.33) | 43.2 (13.15) | 39.8 (13.74) | 44.4 (13.79) | 43.3 (13.82) | 46.7 (14.38) | 40.5 (16.58) | 41.7 (16.16) |
| Female sex, *n* (%) | 12 (54.5) | 37 (52.9) | 49 (53.3) | 6 (60.0) | 15 (44.1) | 21 (47.7) | 5 (31.3) | 22 (43.1) | 27 (40.3) | 1 (14.3) | 17 (60.7) | 18 (51.4) |
| Body weight, median (Q1–Q3) | 63.5 (52.48–74.62) | 72.2 (62.10–84.07) | 70.8 (60.50–82.88) | 71.3 (58.20–78.93) | 72.8 (65.80–86.45) | 72.8 (62.25–85.12) | 68.3 (53.00–76.95) | 70.5 (63.45–89.75) | 70.3 (60.90–87.75) | 83.0 (77.55–84.65) | 66.5 (60.60–81.85) | 69.8 (62.25–84.30) |
| BMI, median (Q1–Q3) | 21.5 (19.52–27.20) | 25.0 (21.75–28.98) | 24.3 (21.19–28.49) | 24.7 (22.29–26.89) | 24.9 (22.14–28.89) | 24.7 (22.03–27.82) | 22.9 (19.60–25.10) | 24.9 (22.42–29.33) | 24.4 (21.64–28.78) | 26.7 (25.50–27.34) | 23.4 (21.19–27.64) | 24.2 (21.82–27.56) |
| Smoking status, *n* (%) |  |  |  |  |  |  |  |  |  |  |  |  |
| Current | 2 (9.1) | 2 (2.9) | 4 (4.3) | 0 | 1 (2.9) | 1 (2.3) | 1 (6.3) | 4 (7.8) | 5 (7.5) | 1 (14.3) | 1 (3.6) | 2 (5.7) |
| Former | 4 (18.2) | 26 (37.1) | 30 (32.6) | 1 (10.0) | 12 (35.3) | 13 (29.5) | 2 (12.5) | 17 (33.3) | 19 (28.4) | 2 (28.6) | 9 (32.1) | 11 (31.4) |
| Never | 16 (72.7) | 42 (60.0) | 58 (63.0) | 9 (90.0) | 21 (61.8) | 30 (68.2) | 13 (81.3) | 30 (58.8) | 43 (64.2) | 4 (57.1) | 18 (64.3) | 22 (62.9) |
| Duration of UC, years, mean (SD) | 9.6 (8.19) | 10.1 (7.20) | 10.0 (7.41) | 7.8 (6.85) | 10.0 (8.58) | 9.5 (8.20) | 6.9 (5.99) | 11.1 (7.11) | 10.1 (7.05) | 10.2 (7.16) | 9.7 (8.00) | 9.8 (7.74) |
| Total Mayo Clinic Score, mean (SD) | 9.0 (1.36) | 9.2 (1.30) | 9.1 (1.31) | 8.8 (1.62) | 9.4 (1.45) | 9.2 (1.49) | 9.4 (1.20) | 9.2 (1.45) | 9.3 (1.39) | 8.9 (0.90) | 9.1 (1.13) | 9.1 (1.08) |
| Mayo endoscopic score of 3, *n* (%) | 19 (86.4) | 50 (71.4) | 69 (75.0) | 7 (70.0) | 25 (73.5) | 32 (72.7) | 12 (75.0) | 35 (68.6) | 47 (70.1) | 6 (85.7) | 20 (71.4) | 26 (74.3) |
| C-reactive protein, mg/L, mean (SD) | 5.4 (6.47) | 11.5 (15.12) | 10.0 (13.78) | 13.1 (11.53) | 10.0 (12.15) | 10.7 (11.96) | 5.7 (7.98) | 13.2 (24.41) | 11.4 (21.83) | 14.3 (18.37) | 7.8 (7.92) | 9.1 (10.79) |
| Faecal calprotectin, μg/g mean (SD) | 1712.2 (1466.08) | 3420.3 (4753.37) | 3017.2 (4268.85) | 2456.0 (4700.54) | 3079.5 (4221.04) | 2949.0 (4274.77) | 2366.4 (2081.26) | 1947.0 (2009.42) | 2042.3 (2017.50) | 1647.1 (2279.38) | 2014.5 (2576.30) | 1934.1 (2483.54) |
| Concomitant use of systemic corticosteroids only, *n* (%) | 0 | 32 (45.7) | 32 (34.8) | 0 | 17 (50.0) | 17 (38.6) | 0 | 30 (58.8) | 30 (44.8) | 0 | 14 (50.0) | 14 (40.0) |
| Concomitant use of IMs only, *n* (%) | 13 (59.1) | 2 (2.9) | 15 (16.3) | 5 (50.0) | 0 | 5 (11.4) | 8 (50.0) | 0 | 8 (11.9) | 3 (42.9) | 0 | 3 (8.6) |
| Concomitant use of systemic corticosteroids and IMs, *n* (%) | 9 (40.9) | 1 (1.4) | 10 (10.9) | 5 (50.0) | 1 (2.9) | 6 (13.6) | 8 (50.0) | 0 | 8 (11.9) | 4 (57.1) | 0 | 4 (11.4) |
| Prednisone-equivalent dose (mg/day), median (Q1–Q3) | 15.0 (10.00 – 20.00) | 15.0 (10.00–20.00) | 15.0 (10.00–20.00) | 10.0 (10.00–10.00) | 20.0 (10.62–27.50) | 20.0 (10.00–22.50) | 20.0 (18.75–21.25) | 20.0 (10.00–20.00) | 20.0 (10.00–20.00) | 20.0 (16.25–20.00) | 17.5 (13.12–20.00) | 20.0 (13.12–20.00) |
| Concomitant use of 5-ASA, *n* (%) | 16 (72.7) | 43 (61.4) | 59 (64.1) | 6 (60.0) | 18 (52.9) | 24 (54.5) | 13 (81.3) | 28 (54.9) | 41 (61.2) | 6 (85.7) | 16 (57.1) | 22 (62.9) |
| Number of prior biologic agents used, *n* (%) |  |  |  |  |  |  |  |  |  |  |  |  |
| 0 | 0 | 1 (1.4) | 1 (1.1) | 0 | 1 (2.9) | 1 (2.3) | 0 | 0 | 0 | 1 (14.3) | 1 (3.6) | 2 (5.7) |
| 1 | 12 (54.5) | 24 (34.3) | 36 (39.1) | 7 (70.0) | 9 (26.5) | 16 (36.4) | 10 (62.5) | 18 (35.3) | 28 (41.8) | 0 | 8 (28.6) | 8 (22.9) |
| 2 | 5 (22.7) | 26 (37.1) | 31 (33.7) | 0 | 10 (29.4) | 10 (22.7) | 3 (18.8) | 18 (35.3) | 21 (31.3) | 3 (42.9) | 11 (39.3) | 14 (40.0) |
| 3 | 5 (22.7) | 19 (27.1) | 24 (26.1) | 3 (30.0) | 14 (41.2) | 17 (38.6) | 3 (18.8) | 15 (29.4) | 18 (26.9) | 3 (42.9) | 8 (28.6) | 11 (31.4) |
| Prior use of at least 1 TNF antagonist, *n* (%) | 20 (90.9) | 63 (90.0) | 83 (90.2) | 10 (100.0) | 32 (94.1) | 42 (95.5) | 14 (87.5) | 48 (94.1) | 62 (92.5) | 6 (85.7) | 24 (85.7) | 30 (85.7) |
| Prior use of vedolizumab, *n* (%) | 8 (36.4) | 40 (57.1) | 48 (52.2) | 3 (30.0) | 20 (58.8) | 23 (52.3) | 6 (37.5) | 24 (47.1) | 30 (44.8) | 3 (42.9) | 12 (42.9) | 15 (42.9) |
| Prior use of at least 1 TNF antagonist and vedolizumab, *n* (%) | 6 (27.3) | 34 (48.6) | 40 (43.5) | 3 (30.0) | 19 (55.9) | 22 (50.0) | 4 (25.0) | 21 (41.2) | 25 (37.3) | 3 (42.9) | 9 (32.1) | 12 (34.3) |
| Prior failure of at least 1 TNF antagonist, *n* (%) | 16 (72.7) | 59 (84.3) | 75 (81.5) | 6 (60.0) | 32 (94.1) | 38 (86.4) | 14 (87.5) | 42 (82.4) | 56 (83.6) | 6 (85.7) | 22 (78.6) | 28 (80.0) |
| Prior failure of vedolizumab, *n* (%) | 7 (31.8) | 33 (47.1) | 40 (43.5) | 3 (30.0) | 18 (52.9) | 21 (47.7) | 4 (25.0) | 22 (43.1) | 26 (38.8) | 3 (42.9) | 10 (35.7) | 13 (37.1) |

5-ASA, 5-aminosalicylic acid; BMI, body mass index; FIL, filgotinib; IM, immunomodulator; PBO, placebo; Q, quartile; SD, standard deviation; TNF, tumour necrosis factor; UC, ulcerative colitis.

^a^+IM includes azathioprine, 6-mercaptopurine, or methotrexate.

**Supplementary Table 3.** Concomitant IM use in induction studies A and B.

|  | **Induction study A:**  **biologic-naive (*N* = 196)** | | | **Induction study B:**  **biologic-experienced (*N* = 156)** | | |
| --- | --- | --- | --- | --- | --- | --- |
|  | **PBO** | **FIL 100 mg** | **FIL 200 mg** | **PBO** | **FIL 100 mg** | **FIL 200 mg** |
| AZA, *n* | 39 | 73 | 61 | 24 | 47 | 44 |
| MP, *n* | 2 | 4 | 10 | 3 | 7 | 12 |
| Methotrexate, *n* | 0 | 5 | 2 | 5 | 8 | 5 |
| Methotrexate sodium, *n* | 0 | 0 | 0 | 0 | 0 | 1 |

AZA, azathioprine; FIL, filgotinib; IM, immunomodulator; MP, 6-mercaptopurine; PBO, placebo.

**Supplementary Table 4.** Concomitant IM use in the maintenance study.

|  | **Induction study A:**  **biologic-naive (*N* = 90)** | | | | **Induction study B:**  **biologic-experienced (*N* = 55)** | | | |
| --- | --- | --- | --- | --- | --- | --- | --- | --- |
|  | **PBO (from FIL100 mg)** | **PBO (from FIL 200 mg)** | **FIL 100 mg (from FIL 100 mg)** | **FIL 200 mg (from FIL 200 mg)** | **PBO from (FIL100 mg)** | **PBO (from FIL 200 mg)** | **FIL 100 mg (from FIL 100 mg)** | **FIL 200 mg (from FIL 200 mg)** |
| AZA, *n* | 13 | 15 | 22 | 26 | 5 | 8 | 14 | 16 |
| MP, *n* | 1 | 2 | 3 | 4 | 0 | 1 | 1 | 4 |
| Methotrexate, *n* | 2 | 0 | 1 | 1 | 2 | 1 | 1 | 2 |

AZA, azathioprine; FIL, filgotinib; IM, immunomodulator; MP, 6-mercaptopurine; PBO, placebo.

**Supplementary Table 5.** Hazard ratios for disease worsening in the maintenance study with and without concomitant IM use in patients treated with filgotinib.

| Concomitant IM use | **FIL 200 mg versus PBO** | | | **FIL 100 mg versus PBO** | | |
| --- | --- | --- | --- | --- | --- | --- |
|  | **HR** | **95% CI** | ***P* value** | **HR** | **95% CI** | ***P* value** |
| +IM | 0.530 | 0.24, 1.18 | 0.120 | 1.017 | 0.52, 2.19 | 0.850 |
| −IM | 0.230 | 0.15, 0.37 | < 0.001 | 0.490 | 0.33, 0.72 | < 0.001 |

CI, confidence interval; FIL, filgotinib; HR, hazard ratio; IM, immunomodulator; PBO, placebo.

**Supplementary Table 6.** Summary of treatment-emergent leukopenia AEs at week 10 by treatment group and concomitant IM use.

| **Induction** | **FIL 200 mg**  **(*N* = 507)** | | **FIL 100 mg**  **(*N* = 562)** | | **PBO**  **(*N* = 279)** | |
| --- | --- | --- | --- | --- | --- | --- |
| **AEs, *n* (%)** | **+IM**^a^  **(*n* = 135)** | **−IM**  **(*n* = 372)** | **+IM**^a^  **(*n* = 144)** | **−IM**  **(*n* = 418)** | **+IM**^a^  **(*n* = 73)** | **−IM**  **(*n* = 206)** |
| Any leukopenia | 4 | 2 | 2 | 1 | 1 | 0 |

AE, adverse event; FIL, filgotinib; IM, immunomodulator; PBO, placebo.

^a^+IM includes azathioprine, 6-mercaptopurine, or methotrexate.

**Supplementary Table 7.** Summary of treatment-emergent leukopenia AEs at week 58 by treatment group and concomitant IM use.

| **Induction** | **FIL 200 mg** | | | | **FIL 100 mg** | | | | **PBO** | |
| --- | --- | --- | --- | --- | --- | --- | --- | --- | --- | --- |
| **Maintenance** | **FIL 200 mg** | | **PBO** | | **FIL 100 mg** | | **PBO** | | **PBO** | |
| **AEs, *n*** | **+IM**^a^  **(*n* = 54)** | **−IM**  **(*n* = 148)** | **+IM**^a^  **(*n* = 27)** | **−IM**  **(*n* *=* 72)** | **+IM**^a^  **(*n* = 44)** | **−IM**  **(*n* = 135)** | **+IM**^a^  **(*n* = 24)** | **−IM**  **(*n* = 67)** | **+IM**^a^  **(*n* = 30)** | **−IM**  **(*n* = 63)** |
| Any leukopenia | 1 | 0 | 1 | 0 | 1 | 1 | 0 | 0 | 1 | 0 |

AE, adverse event; FIL, filgotinib; IM, immunomodulator; PBO, placebo.

^a^+IM includes azathioprine, 6-mercaptopurine, or methotrexate.

**Supplementary Table 8.** Summary of treatment-emergent AEs at week 10 by treatment group and concomitant IM and corticosteroid use

|  | **FIL 200** | | | | **FIL 100** | | | | **PBO** | | | |
| --- | --- | --- | --- | --- | --- | --- | --- | --- | --- | --- | --- | --- |
|  | **+IM^a^** | | **–IM** | | **+IM^a^** | | **–IM** | | **+IM^a^** | | **–IM** | |
| **AEs, *n* (%)** | **+CS**  **(*n* = 48)** | **–CS**  **(*n* = 87)** | **+CS**  **(*n* = 148)** | **–CS**  **(*n* = 224)** | **+CS**  **(*n* = 47)** | **–CS**  **(*n* = 97)** | **+CS**  **(*n* = 170)** | **–CS**  **(n = 248)** | **+CS**  **(*n* = 19)** | **–CS**  **(*n* = 54)** | **+CS**  **(*n* = 85)** | **–CS**  **(*n* = 121)** |
| Any AE | 25 (52.1) | 45 (51.7) | 85 (57.4) | 117 (52.2) | 23 (48.9) | 50 (51.5) | 101 (59.4) | 109 (44.0) | 9 (47.3) | 37 (68.5) | 50 (58.8) | 61 (50.4) |
| Any SAE | 1 (2.1) | 0 (0.0) | 12 (8.1) | 9 (4.0) | 4 (8.5) | 4 (4.1) | 11 (6.5) | 9 (3.6) | 0 (0.0) | 3 (5.6) | 5 (5.9) | 5 (4.1) |
| Any infections and infestations | 11 (22.9) | 11 (12.6) | 30 (20.3) | 40 (17.9) | 4 (8.5) | 14 (14.4) | 32 (18.8) | 32 (12.9) | 5 (26.3) | 10 (18.5) | 12 (14.1) | 12 (9.9) |

AE, adverse event; CS, corticosteroids; FIL, filgotinib; IM, immunomodulator; PBO, placebo; SAE, serious adverse event.

^a^+IM includes azathioprine, 6-mercaptopurine, or methotrexate.

**Supplementary Figure 1.** SELECTION study design and *post hoc* analyses.


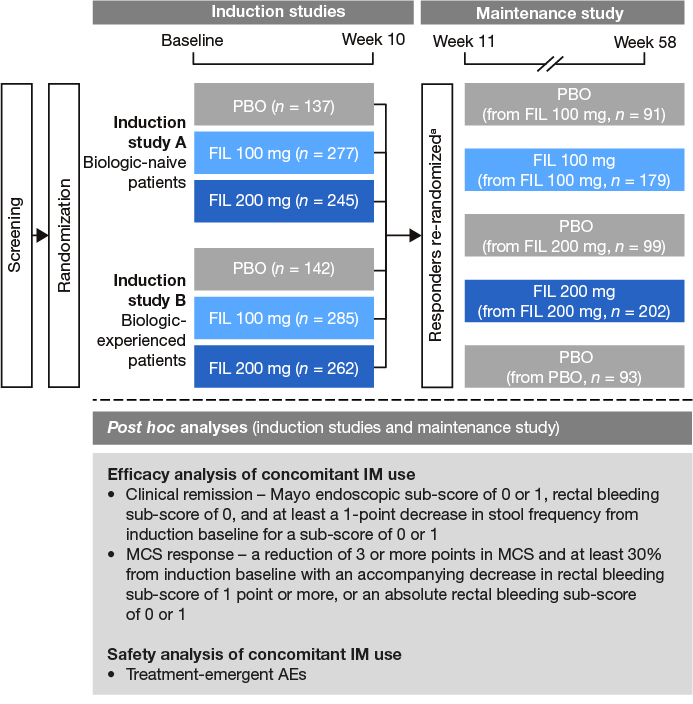


AE, adverse event; FIL, filgotinib; IM, immunomodulator; MCS, Mayo Clinic Score; PBO, placebo.

^a^Patients achieving clinical remission or MCS response to FIL induction therapy were re-randomized 2:1 to continue their FIL induction dose or to receive PBO from week 11 to week 58. Patients achieving clinical remission or MCS response to PBO continued to receive PBO.

**Supplementary Figure 2** Kaplan–Meier curves for time to protocol-specific disease worsening during the maintenance study with and without concomitant IM use in patients treated with filgotinib 100 mg [A] and all filgotinib groups combined [B].


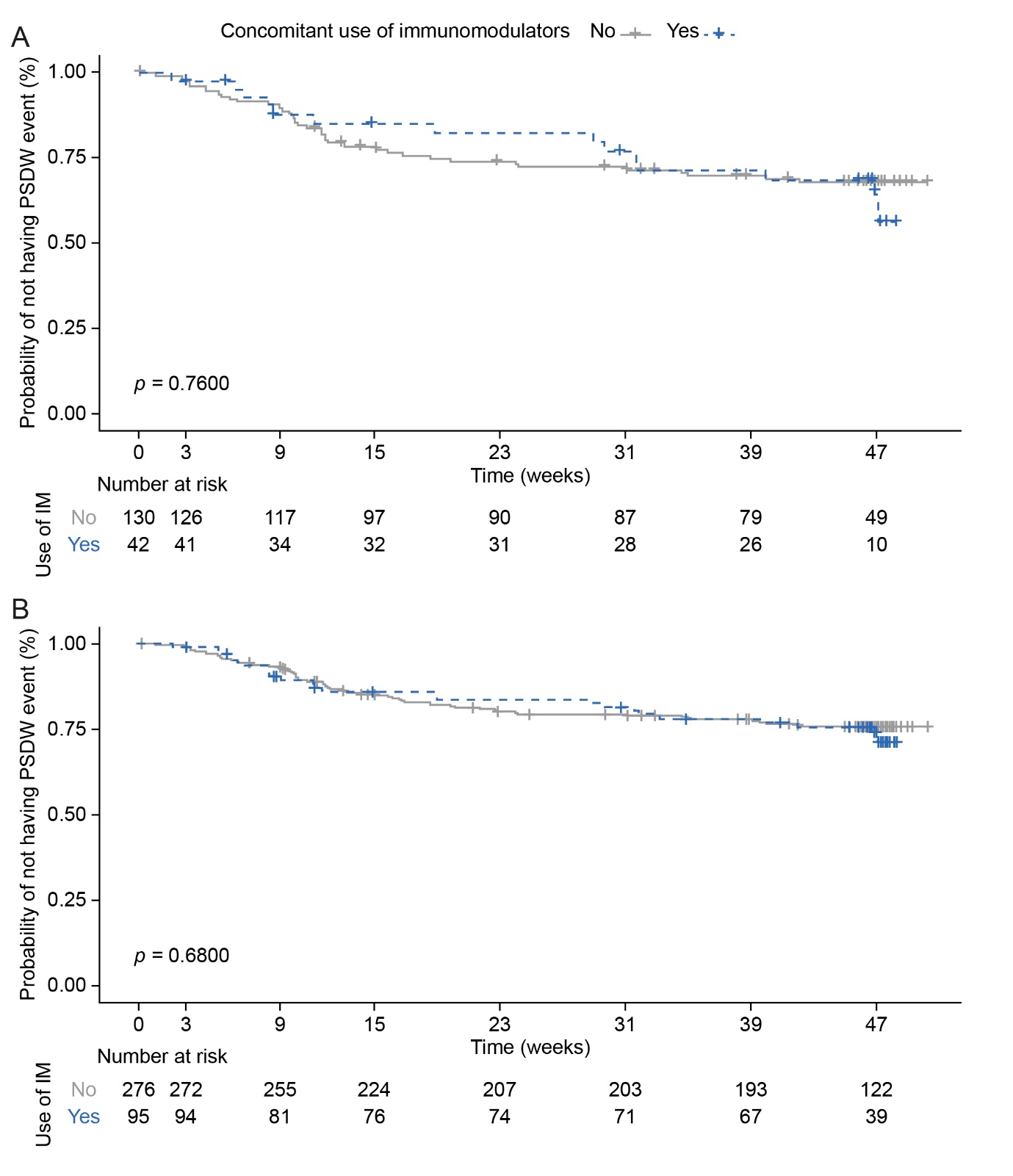


IM, immunomodulators; PSWD, protocol-specified disease worsening.
